# Sclerosing angiomatoid nodular transformation of the spleen: multimodality imaging features and literature review

**DOI:** 10.1186/s12880-023-01008-3

**Published:** 2023-04-06

**Authors:** Ning-Xin Chen, Ming-Liang Wang, Hai-Xing Wang, Meng-Su Zeng

**Affiliations:** 1 Department of Radiology, The Second Affiliated Hospital of Fujian Medical University, No. 34 Zhongshan Bei Road, Licheng District, Quanzhou, Fujian China; 2 Department of Radiology, Zhongshan Hospital, Fudan University, No. 180 Fenglin Road, Xuhui District, Shanghai, 200032 China; 3 Department of Pathology, Zhongshan Hospital, Fudan University, No. 180 Fenglin Road, Xuhui District, Shanghai, 200032 China

**Keywords:** Sclerosing angiomatoid nodular transformation, Computed tomography imaging(CT), Magnetic resonance imaging (MRI)

## Abstract

**Objective:**

The purpose of this study was to evaluate the CT and MRI findings, clinicopathologic features, and differential diagnosis of Sclerosing angiomatoid nodular transformation (SANT).

**Methods and materials:**

Seven men and seven women with pathological diagnoses of SANT were included in this retrospect study. Patients underwent at least one radiological examination before surgery. The number, shape, margin, size, attenuation, signal intensity, homogeneity, and enhancing pattern of the lesion were evaluated by two abdominal radiologists independently. Immunohistochemistry reports were available for 11 patients. The immunoreactivity to the vascular markers CD8, CD31, and CD34 was assessed.

**Results:**

The 14 SANT patients (7 men, 7 women; mean age, 43.5 years; age range, 24–56 years) presented with a single lesion and showed no specific clinical symptoms. Among 14 patients, 12 patients underwent MR scan, 5 patients underwent CT scan and 3 patients underwent PET-CT. On CT, all 5 lesions showed hypodensity on non-contrast images and spoke-wheel enhancing pattern after contrast administration, and calcification was observed. On T2WI, 10 cases(83.3%)showed hypointensity and 2 cases (16.7%) showed hyperintensity with central hypointensity. On T1WI, 10 cases (83.3%) were isointense and 2 cases (16.7%) were slightly hypointense. 10 cases (83.3%) showed hypointensity on DWI and 2 cases (16.7%) showed slightly hyperintensity on DWI. After contrast administration, all 12 lesions showed progressive enhancement. 18 F-fluorodeoxyglucose (FDG) uptake in the tumor was seen in all three cases that underwent PET-CT. The maximum standardized uptake value (SUVmax) was 4.5, 5.1, and 3.8 respectively.

**Results:**

Apart from the progressive spoke-wheel enhancing pattern, DWI and ADC findings will add value to the diagnosis of SANT.

## Introduction

Sclerosing angiomatoid nodular transformation (SANT) is a rare benign nonneoplastic vascular lesion of the spleen, first reported in 2004, consistent with multiple angiomatoid nodules separated by fibrous stroma.[1] Patients with SANT usually were asymptomatic and identified incidentally on imaging. SANT does not appear to be related to age or gender, although some reports have reported a middle-aged female predominance. [2, 3] SANT can only be correctly diagnosed with a tissue sample for histopathology and immunohistochemistry evaluation.[4].

Since 2004, many reports described the pathology of SANT, however, the reports about imaging characteristics have been limited to case reports. Because SANT has varying growth patterns, Radiologists would misdiagnose as other splenic tumors due to lack of knowledge regarding SANT. Therefore, it’s important to know the characteristic imaging finding to distinguish it from other splenic tumors.

Although computed tomography (CT) imaging, magnetic resonance imaging (MRI) 18 F-fluorodeoxyglucose (FDG) positron emission tomography (PET)/CT findings feature of SANT have been reported.[5–7] Nowadays, DWI has been widely used in Abdominal applications. With ADC map, DWI can be qualitatively and quantitatively assessed and help for differentiating benign from malignant tumors in abdominal MRI examinations. However, imaging characterization of splenic lesions with DWI is challenging due to the spleen has the greatest degree of nonpathological impeded diffusion in all solid abdominal organs. Because of the rich blood supply of the spleen, splenic biopsy may be difficult to perform, which may lead to unnecessary splenectomy. Also due to the low incidence of SANT, the evaluation of the value of DWI and ADC map findings in SANT is only mentioned in few literature [8–12]. In this study, we present multimodality imaging appearance of 14 SANTs, including 12 cases with DWI and ADC map findings and evaluate the added value of them.

## Materials and methods

### Patients

This retrospective study was approved by our institutional review board. Informed consent was not required. We retrospectively searched the database between January 2017 and November 2019. The inclusion criteria were as follows: (1) Certain pathological diagnosis of SANT; (2) Pre-operative examination including CT, MRI or PET-CT. Finally, seven men and seven women were included in our study with an average age of 43.5 (rang from 24 to 56). One patient complained of right upper quadrant pain, two patients discovered the lesions incidentally during routine imaging evaluation of hepatocellular carcinoma and renal carcinoma. Other patients were asymptomatic. All patients underwent splenectomy.

### Imaging methods

Imaging was performed at different CT and MR systems. 12 patients underwent MR scan. 5 patients underwent CT scan. 3 patients underwent PET-CT. Among 14 patients, one underwent CT, MRI and PET-CT scan, 2 underwent CT and MRI scan and 2 underwent MRI and PET-CT scan. All CT scans included unenhanced (before contrast administration), arterial phase (25–30 s), and portal venous phase (70–80 s). 80 to 120 mL of iohexol (Omnipaque 300; Amersham, Shanghai, China) was injected into the antecubital vein using an 18-gauge angiographic catheter by using a power injector (LF CT 9000; Liebel-Flarsheim, Cincinnati, OH) at a flow rate of 2.5 to 3.0 mL/s. Saline was injected at the same flow rate. The parameters of the CT scan were as follows: tube potential, 120 kV; section thickness, 5 mm; reconstruction interval, 5 mm. All MRI scans included transverse respiratory-navigated T2-weighted fat-suppressed turbo spin-echo sequence [repetition time (TR)/echo time (TE) = 3500/84 ms; section thickness, 7 mm; intersection gap, 2.1 mm; field of view optimized to patients’ body habitus, 320 × 320–380 × 380 mm; matrix, 194 × 256] and T1-weighted in-phase/out-phase [TR/TE = 6.8/2.35 (in-phase), 4.75 (opposed-phase) ms; section thickness,7 mm; intersection gap, 2.1 mm; field of view optimized to patients’ body habitus, 320 × 320–380 × 380 mm; matrix, 194 × 256], transverse breath-hold single-shot DWI with 2 b values (0 and 500 s/mm^2^) and dynamic breath-hold fat-saturated gadolinium-enhanced T1-weighted sequences with arterial(20-25s), pancreatic(35-40s), portal venous(55-60s), and delayed phases(180 s). Gadopentetate dimeglumine (Magnevist, Bayer Schering Pharma, Berlin, Germany), was administered at a dose of 0.1 mmol/kg and at a rate of 2 ml/s followed by using a power injector (Spectris; Medrad, Pittsburgh, PA, USA). 20-ml saline was injected at the same flow rate. PET-CT scanned 60 min and 120 to 150 min after FDG administration. The CT scan was done according to a standard protocol at 120–140 kVp and 180–300 mA at 2 min per field of view, and a 3.75 mm section thickness to match the PET section thickness.18 F-FDG 3.70-5.55MBq/kg was administered intravenously according to body weight.

### Imaging analysis

Two abdominal radiologists with 10 and 15 years of experience reviewed the images independently. Final interpretation was reached in consensus. The number, shape, margin, size, attenuation, signal intensity, homogeneity and enhancing pattern of the lesion was evaluated. The density and signal intensity were recorded as hypodensity/hypointensity, isodensity/isointensity and hyperdensity/hyperintensity compared with surrounding splenic parenchyma. The presence of calcification, necrosis, cystic change or hemorrhage was also recorded. For PET-CT, the maximum standardized uptake value (SUV) of the lesion during the early and delayed phases was measured.

### Pathologic analysis

A pathologist reviewed pathological reports and slides to confirm or establish the diagnosis of SANT and determined the histologic presence. Immunohistochemistry reports were available for 11 patients. The immunoreactivity to the vascular markers CD8, CD31, and CD34 was assessed.

## Results

### Clinical and pathological features

**Fig. 1:**
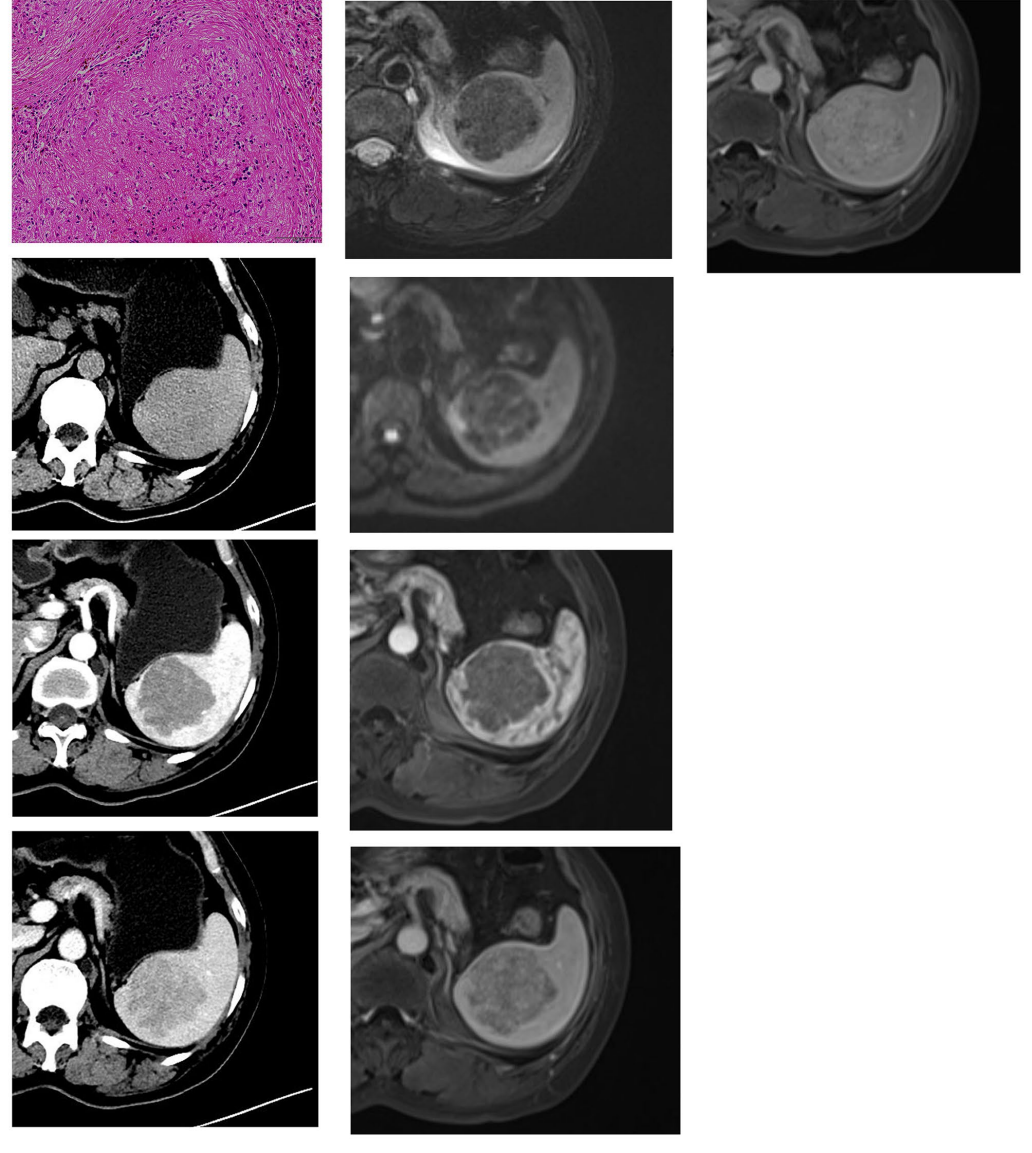
An elderly woman with a history of anemia. Microscopically, the splenic lesion consisted of variably sized nodules surrounded by variable fibrous bands (H&E, original magnification×200). There was proliferation of both collagen fiber (B) and multiple micro-vessels, with inflammatory cell infiltration(1 A). Unenhanced axial CT image (1B) shows a hypodense lesion in spleen. The lesion shows heterogeneous enhancement on arterial phase(1 C) and portal phase(1D). T2-weighted images(1E) and DWI(1 F) show a hypointensity lesion with more hypointense scars in the center. After contrast administration, the lesion shows less contrast-enhanced than the spleen parenchyma in arterial phase(1G) and portal venous phase(1 H). The lesion shows continued progressive enhancement on delayed phase(1I)

The clinical characteristics of these 14 cases are presented in Table 1. A solitary splenic tumor was seen in all cases. Generally, the resected specimen showed the lesions well-circumscribed appearance with central gray-white stellate fibrous scar. The size of the tumor was ranged from 4 × 2 × 2 cm to 15 × 10 × 2 cm. These lesions were composed of multiple vascular nodules separated by interspersed bands of fibrous tissue (Fig. 1A). Calcification was found in 2 cases; hemorrhage was found in 4 cases. 4 cases showed histocytes and inflammatory cells in the stroma. 11 cases had an immunohistochemistry report. All 11 cases were positive for CD31 and CD34. CD8 and CD86 positive expression was presented in 6 and 7 cases respectively.

**Table 1:** Clinical and pathological characteristics

Case No.	sex	Gross size	Boundary	texture	calcification	hemorrahge	Capsule
1	F	9 × 7.5 × 7 cm	Clear	Hard	-	-	-
2	M	4 × 4 × 3.5 cm	Clear	Moderate	-	-	-
3	F	4 × 4 × 3 cm	Unclear	Moderate	+	+	-
4	M	4.5 × 4 × 3 cm	Unclear	hard	-	-	-
5	M	4 × 2 × 2 cm	Unclear	Moderate	-	-	-
6	M	8.5 × 8 × 7 cm	Clear	Moderate	-	-	-
7	F	4 × 3.5 × 3 cm	Unclear	Moderate	+	-	-
8	M	6.5 × 6 × 4 cm	Clear	Soft	-	-	+
9	F	7 × 6.5 × 5 cm	Clear	Hard	-	-	-
10	F	6 × 4 × 4 cm	Unclear	Moderate	-	+	-
11	M	6 × 4.5 × 4.5 cm	Clear	Hard	-	-	-
12	F	4.5 × 4 × 3.5 cm	Unclear	Hard	-	+	-
13	M	15 × 10 × 2 cm	Unclear	Hard	-	+	+
14	F	7 × 5 × 3 cm	Unclear	Hard	-	-	-

### Imaging features

All the lesions are well-defined and solitary with a lobulated (n = 2) or smooth (n = 12) margin in our study. Imaging features are presented in Table 2.

**Table 2:** Imaging features

		Imaging features							
Case No.	density		T2WI	T1WI	DWI	ADC map	Enhancing pattern	PET-CT	SUV(early and delayed phase)
1	Hypodensity		Heterogeneous hyperintensity	Isointensity	Heterogenous hypointensity	Hyperintensity	Spoke-wheel, progressive, centripetal	Uptake	4.5/4.0
2	N/A		Slightly hypointensity	Isointensity	Heterogenous hypointensity	Hyperintensity	Spoke-wheel, progressive, centripetal	Uptake with calcification on CT	5.1
3	N/A		Slightly hyperintensity	Hypointensity	Hypointensity	Hyperintensity	Progressive eccentric	N/A	N/A
4	N/A		Hypointesity	Hypointensity	Hypointensity	Hyperintensity	Progressive circumferential	N/A	N/A
5	N/A		Hypointensity	Isointensuty	Hypointensity	Isointensity	Progressive	N/A	N/A
6	N/A		Hyperintensity	Hypointensity	Slightly hyperintensity	Hyperintensity	Spoke-wheel, progressive, centripetal	N/A	N/A
7	N/A		Hypointensity	Isointensity	Hypointensity	Hyperintensity	Spoke-wheel, progressive, centripetal	Uptake with calcification on CT	2.8/3.8
8	N/A		Isointensity	Isointensity	Hyperintensity	Hyperintensity	Progressive	N/A	N/A
9	N/A		Hypointensity	Isointensity	Hypointensity	Hyperintensity	Progressive	N/A	N/A
10	Hypodensity		Hypointensity	Isointensity	Hypointensity	Hypointensity	Progressive	N/A	N/A
11	Hypodensity		Hypointensity	Isointensity	Hypointensity	Isointensity	Spoke-wheel, progressive, centripetal	N/A	N/A
12	N/A		Hypointensity	Isointensity	Hypointensity	Hyperintensity	Progressive	N/A	N/A
13	Hypodensity		N/A	N/A	N/A	N/A	Progressive	N/A	N/A
14	Hypodensity		N/A	N/A	N/A	N/A	Progressive	N/A	N/A

N/A, not available; SUV, standardized uptake value

**Fig. 2:**
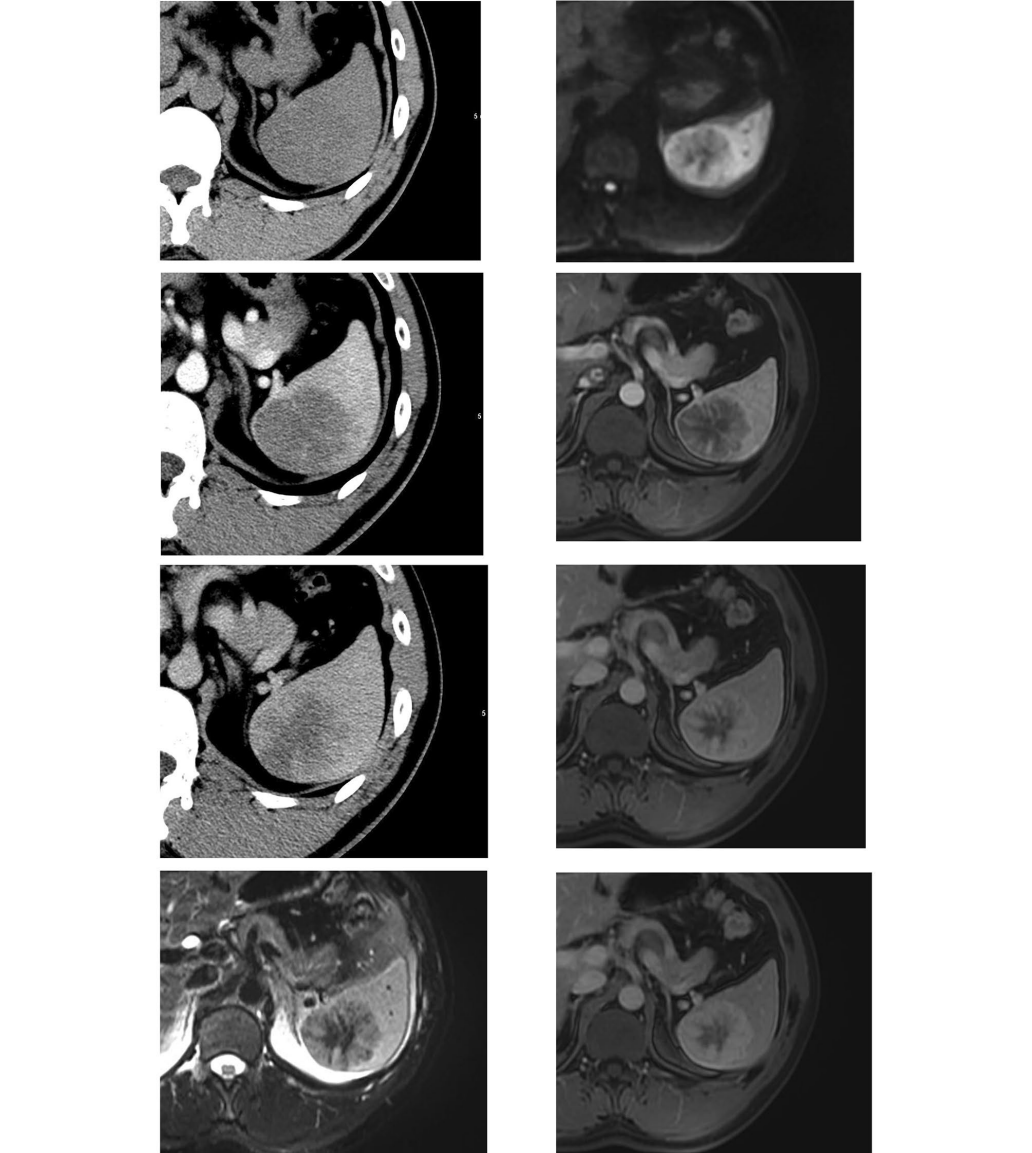
A middle-aged male with no specific clinical symptoms was diagnosed with SANT. Unenhanced axial CT image (2 A) shows a hypodense lesion in spleen. On contrast-enhanced CT images(arterial phase:2B and portal phase:2 C), the lesion shows progressive and centripetal enhancement. The lesion shows hypointensity on T2WI(2D) and DWI(2E), with central more hypointense scars. After contrast administration, enhancement with the pattern of spoke-wheel is seen during arterial phase(2 F), portal venous phase(2G) and delayed phase(2 H)

On CT, all 5 lesions showed hypodensity on non-contrast images (Figs. 1B and 2A) and progressive radial heterogeneous enhancement after contrast administration (Figs. 1C and D and 2B and C). Intralesional calcification was not seen in all 5 lesions.

The signal intensity was generally heterogeneous on both non-contrast and contrast images. Among 12 cases, 10 cases(83.3%)showed hypointensity on T2weighted imaging (Figs. 1E and 2D), and 2 cases (16.7%) showed hyperintentisy with central hypointensity. On T1 weighted imaging, 10 cases (83.3%) were isointense and 2 cases (16.7%) were slightly hypointense. One case dropped signal intensity on in- phase on T1 weighted imaging. 10 cases (83.3%) showed hypointensity on Diffusion weighted imaging (Figs. 1F and 2E) and 2 cases (16.7%) showed slightly hyperintensity on DWI. On the ADC map, only one case (8.3%) showed hypointensity, other 11 cases (91.7%) showed hyperintensity or isointensity. After contrast administration, all 12 lesions showed progressive enhancement(Fig. 1G, H, I). The centripetal “spoke-wheel” pattern with central hypointense scar was observed in 5 (41.6%) cases (Fig. 2F, G, H). One case (8.3%) showed progressive eccentric enhancement. One showed (8.3%) progressive circumferential enhancement. One (8.3%) showed marked enhancement on arterial phase and remained hyperintensity relative to the spleen. Cystic change or necrosis was absent in all 12 patients.

18 F-fluorodeoxyglucose (FDG) uptake in the tumor was seen in all three cases that underwent PET-CT. The maximum standardized uptake values (SUVmax) were 4.5, 5.1, and 3.8 respectively.

## Discussion

In addition to the typical “spoke-wheel” performance on enhanced images, our study demonstrated that DWI and ADC findings could raise the diagnostic performance for the diagnosis of SANT. SANT is known as a benign vascular neoplasm of uncertain etiology described first in 2004. Before that, literatures have recently reinterpreted the lesion as splenic hamartoma, cord capillary hemangioma, splenic hemangioendothelioma. The etiology may be the ultimate ending of a variety of benign splenic conditions, such as inflammatory pseudotumor and hamartoma.[1] [13] The gross pathologic feature of SANT is a well-defined mass composed of numerous angiomatoid nodules separated by fibrosclerotic stroma that often form a central stellate scar. Three types of blood vessels are observed including cord capillaries, splenic sinusoids, and small veins. Their staining patterns with various markers including CD34, CD21, and CD8 can be identified. CD68-positive staining pattern is also helpful.[14] The composition of multiple types of blood vessels, the particular architectural features and the expression of immunophenotype differs SANT from other splenic vascular neoplasms.[1] It was reported as an asymptomatic lesion with slight female predominance. In our study, 11(78.6%) of 14 cases were asymptomatic and the other 3 cases found the neoplasm occasionally or during the treatment of other diseases.

A few reports with single case described the CT and MRI features of SANT. These literature focus on conventional T1WI, T2WI and enhanced images. SANT is hypointense on non-contrast CT imaging with progressive enhancement after contrast administration. This progressive centripetal in a radiating fashion can be observed more easily on contrast MR imaging and was called the “spoke-wheel” pattern. [15]. The“spoke-wheel”pattern, considering as an typical imaging feature, had low sensitivity, which was presented in 5 cases (41.7%) in our study and 11 cases(48%) in a recent review study.[16] Progressive eccentric and circumferential enhancement were observed in 2 cases (16.7%) in our study. Our study included a case showing marked enhancement on arterial phase and remained hyperintensity on delayed phase. Lewis et al. suggested that may be due to continued enhancement of the angiomatous nodules with delayed enhancement of the fibrous tissue. [17]

On T2WI, most of SANTs are hypointense with more central hypointense due to their fibrous composition with a greater proportion of fibrous content. Some SANTs showed hyperintense at the periphery with hypointensity at the center, and have hypointense radiation bands, corresponding to a central stellate fibrous stroma with fibrous septa.[5]. Signal intensity decrease can be observed on in-phase of T1WI, indicating the presence of hemosiderin deposition due to old hemorrhage. In our study, only one case presented signal decrease on in-phase. Although our pathologic review observed hemorrhage in four cases, no hyperintensity on T1WI or hyperdensity on unenhanced CT was presented in our study. There may be two possibilities. We hypothesize one of them was the hyperintensity on T1WI or hyperdensity on unenhanced CT covered by hypointensity/hypodensity formed by massive fibrosis. Another was the amount of hemorrhage is not enough for definite hyperintensity/hyperdensity. [9]

Due to the recent review literature, about 51.6% of SANT presented hypointensity on T2WI, showing a better sensitivity than “spoken-wheel” patten(48%).[16] A more significant signal decrease could be seen on DWI and T2WI because of a more significant susceptibility effect of DWI. Therefore, DWI may be more effective than enhanced CT, T2WI and dynamic MRI in diagnosing SANT, with features of hypointensity on DWI and hyperintensity on ADC map in few literature[8–10]. Diffusion weighted imaging is sensitive to detect malignant tumors due to the restricted or impeded diffusion in malignance with high cellularity. However, it remained a challenge to distinguish benign splenic tumors from malignant splenic tumors because the normal spleen has the highest restricted diffusion in all solid abdominal organs.[18, 19] Yoshimura et al. suggested that multinodular hyperintense areas and fibrotic hypointense areas can be easily observed on DWI, similar to our study, may be the characteristic feature of SANT. The central hypointensity on DWI may be due to the deposition of hemosiderin substance. Choi et al. reported that diffusion restriction was more reliable than single DWI signal in differentiating the malignant from benign splenic lesions. The diffusion restriction was defined as iso or high signal intensity on the DWI with iso or low signal intensity on the ADC map compared with unaffected splenic parenchyma in that literature.[20] Our study also found most SANTs(83.3%)were absence of diffusion restriction, suggesting benignity. However, sometimes SANT with iron deposition can cause a susceptibility artifact showing hypointensity on both DWI and ADC map, with a signal decrease on in-phase image. [9] This feature was observed in one patient in our study.

SANT was not expected to have FDG accumulation as a benign lesion. However, previous studies, including our study, indicated SANT showing hypermetabolic activity on PET-CT. [11, 21] This may be due to the abundance of cells, including hemosiderin-laden macrophages, myofibroblasts, lymphocytes, and plasma cells. [21] The SUVs in our study, however, were higher than the previous reports, with a SUV max of 4.5, 5.1 and 3.8 respectively.

The differential diagnosis of SANT includes other benign lesions and malignant lesions, such as hamartomas, hemangiomas, littoral cell angiomas, metastatic tumors, angiosarcomas, and lymphoma. Although radiologic differential diagnosis is difficult, some features may help confident differentiation. Hemangiomas and hamartomas can be distinguished from SANT by their hyperintensity on T2WI. Absence of splenomegaly and abdominal lymphadenopathy is helpful in distinguishing SANT from lymphoma. Innumerable lesions are typical features of littoral cell angioma, in contrast to SANT, which is mostly solitary. The primary malignancy is helpful in the diagnosis of metastatic lesions in the spleen. Due to its aggressive behavior, angiosarcoma usually has necrotic areas and distant metastasis. [11, 22] Finally, although SANT is a benign tumor with no recurrence or malignant transformation so far, it can increase in size in a follow-up study.[3] Lacking of CT value and ADC value was the limitation in our study. Further studies with ADC values were recommended.

There were several limitations in our study. Firstly, some modalities’ information was incomplete due to the natural characteristic of the retrospective study. Secondly, small samples were included due to the low incidence.

## Conclusion

In summary, although rare and difficult, SANT has some characteristic features helping to distinguish it from other splenic tumors. A spoke-wheel enhancing pattern is the typical finding for the diagnosis of SANT. In addition, with DWI and ADC findings on MRI, the diagnosis will be more reliable.

## Data Availability

The data that support the findings of this study are available from the corresponding author upon reasonable request.
